# Potable Water Recovery for Space Habitation Systems Using Hybrid Life Support Systems: Biological Pretreatment Coupled with Reverse Osmosis for Humidity Condensate Recovery

**DOI:** 10.3390/membranes15070212

**Published:** 2025-07-16

**Authors:** Sunday Adu, William Shane Walker, William Andrew Jackson

**Affiliations:** 1 Department of Civil, Environmental, and Construction Engineering, Texas Tech University, 911 Boston Avenue, Lubbock, TX 79409, USA; sadu@ttu.edu (S.A.); shane.walker@ttu.edu (W.S.W.); 2 Water and the Environment Research (WATER) Center, Texas Tech University, 911 Boston Avenue, Lubbock, TX 79409, USA

**Keywords:** membrane aerated biological reactor (MABR), humidity condensate (HC), space habitation wastewater, life support system, brackish water reverse osmosis (BWRO), international space station (ISS), wastewater recycling

## Abstract

The development of efficient and sustainable water recycling systems is essential for long-term human missions and the establishment of space habitats on the Moon, Mars, and beyond. Humidity condensate (HC) is a low-strength wastewater that is currently recycled on the International Space Station (ISS). The main contaminants in HC are primarily low-molecular-weight organics and ammonia. This has caused operational issues due to microbial growth in the Water Process Assembly (WPA) storage tank as well as failure of downstream systems. In addition, treatment of this wastewater primarily uses adsorptive and exchange media, which must be continually resupplied and represent a significant life-cycle cost. This study demonstrates the integration of a membrane-aerated biological reactor (MABR) for pretreatment and storage of HC, followed by brackish water reverse osmosis (BWRO). Two system configurations were tested: (1) periodic MABR fluid was sent to batch RO operating at 90% water recovery with the RO concentrate sent to a separate waste tank; and (2) periodic MABR fluid was sent to batch RO operating at 90% recovery with the RO concentrate returned to the MABR (accumulating salinity in the MABR). With an external recycle tank (configuration 2), the system produced 2160 L (i.e., 1080 crew-days) of near potable water (dissolved organic carbon (DOC) < 10 mg/L, total nitrogen (TN) < 12 mg/L, total dissolved solids (TDS) < 30 mg/L) with a single membrane (weight of 260 g). When the MABR was used as the RO recycle tank (configuration 1), 1100 L of permeate could be produced on a single membrane; RO permeate quality was slightly better but generally similar to the first configuration even though no brine was wasted during the run. The results suggest that this hybrid system has the potential to significantly enhance the self-sufficiency of space habitats, supporting sustainable extraterrestrial human habitation, as well as reducing current operational problems on the ISS. These systems may also apply to extreme locations such as remote/isolated terrestrial locations, especially in arid and semi-arid regions.

## 1. Introduction

Water is a vital resource in human spaceflight, accounting for approximately 65% of a crew member’s daily mass intake [1]. As human exploration extends farther from Earth to destinations such as the Moon and Mars, the cost and risk associated with water resupply increase significantly. To enable long-term residence in space, the development of reliable and sustainable water recycling systems is essential. NASA has made significant advancements in water treatment technologies that convert reclaimed wastewater into potable water [2,3]. One important source of reclaimable water is humidity condensate, which offers a sustainable input stream for water recovery systems supporting long-duration missions [3,4,5]. On the International Space Station (ISS), humidity condensate is derived from moisture in the cabin atmosphere, primarily produced through human respiration, perspiration, and other evaporative processes [3,6,7]. The environmental control and life support system (ECLSS) manages the collection and purification of this condensate to maintain safe environmental conditions for the crew [8]. Humidity levels are regulated by the temperature and humidity control (THC) subsystems, which utilize condensing heat exchangers to cool cabin air and remove excess water vapor [6,9]. The resulting liquid is separated from the air stream and transferred to the water recovery system (WRS) for further treatment. Effective collection and purification of humidity condensate are essential to water recovery and crew sustainability in microgravity environments [7,9,10,11].

Future extraterrestrial habitats, such as those envisioned on the Moon or Mars, will rely on partial gravity habitation (PGH) systems. These systems are likely to operate with limited or no water resupply, necessitating self-sufficiency through advanced water recycling. Wastewater sources in PGH environments will include humidity condensate, urine, hygiene water, and fluid from showers and laundry. Unlike in microgravity, density-driven processes in partial gravity environments behave similarly to those on Earth, impacting fluid dynamics and treatment strategies. In light of the spatial and resource constraints of spacecraft and extraplanetary habitats, proposed treatment technologies must prioritize minimal footprint and low total system mass [12].

Biological treatment offers a promising approach to stabilizing humidity condensate. It can minimize downstream biofilm formation, reduce reliance on hazardous chemical pretreatments, and lower pH to suppress ammonia volatilization and reduce bicarbonate concentrations that reduce anion exchange media capacity. Membrane-aerated biological reactors (MABRs) are particularly well-suited for this application as they use bubbleless aeration (oxygen diffusion through non-porous membranes), reducing odors and water vapor loss [13,14,15]. MABRs have been extensively studied for habitation wastewater, including treatment of urine (U), greywater (GW), HC, and combined wastewater (U + GW + HC) [7,16,17,18,19]. Reaction rates (10–60 g-C/m[3]-d), were low compared to suspended growth systems, but MABRs have been operated for long periods (~5 years) without solids management systems which simplifies their operation and reduces secondary waste streams. Micro-gravity-compatible MABRs treating HC were able to remove greater than 80–90% of OC and oxidize > 60% of the total ammoniacal nitrogen (TAN) while maintaining a pH < 7 [20,21,22].

Reverse osmosis (RO) has been widely adopted in terrestrial water treatment, especially for desalination, and there is growing interest in potable reuse [23]. As a pressure-driven membrane separation process, RO effectively removes dissolved salts, organic materials, and other impurities, resulting in high-quality, reusable water [24,25]. The integration of biological and membrane-based technologies—known as hybrid wastewater treatment systems—is an emerging strategy for habitation wastewater management [16]. These systems combine the benefits of biological stabilization with the high rejection capacity of membrane separation, offering a compact and efficient solution for closed-loop life support in space environments [16,26]. Previous work has shown that a low-pressure RO system was able to produce near-potable water from the fluid of an MABR treating greywater and process up to 3000 L on a single membrane at 90% recovery [16]. When applied to humidity condensate treatment in space systems, RO significantly enhances water recovery performance.

**Figure 1:**
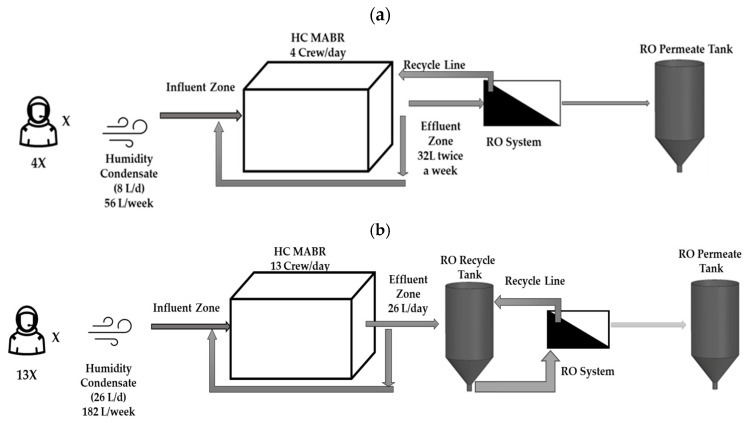
Flow diagram for HC MABR wastewater treatment with BWRO unit: (**a**) RO brine/reject recycled to MABR and (**b**) RO brine/reject recycled to separate RO feed/concentrate recycle tank (adapted from Hooshyari et al. [16]).

In this study, a full-scale hybrid treatment system for humidity condensate was evaluated, as illustrated in Figure 1. The system integrates a variable volume membrane-aerated biological reactor (MABR) for pretreatment of the humidity condensate (HC) with a brackish water reverse osmosis (BWRO) unit (Fresh water Systems at Greenville, SC, USA). Two configurations were assessed to determine system performance in terms of processing capacity, throughput, consumables, and water quality (Figure 1). The MABR continuously received simulated habitation HC, which was the feed to the RO system. In configuration 1 (Figure 1a), the MABR reactor fluid was processed directly from the MABR with the RO concentrate recycled directly back to the MABR. In this configuration no brine was wasted, and rejected species were stored in the MABR, and the system recovered 100% of the HC wastewater. In contrast, configuration 2 (Figure 1b) employed a daily release of MABR fluid to the RO recycle tank, which was processed by the RO in batch mode. The final brine, representing 10% of the total batch volume, was drained for further processing. This study therefore tested both an external RO recycle tank (United States Plastic Corp, Lima City, OH, USA) and the direct use of the MABR as the recycle reservoir, enabling the evaluation of trade-offs in system mass, volume, consumables, and operational efficiency. This analysis provides insight into the application and evaluation of hybrid biological–membrane treatment systems for partial gravity habitats, particularly in managing humidity condensate wastewater streams [16,27].

## 2. Materials and Method

### 2.1. Configuration and Process of the HC MABR and BWRO System

The treatment system is composed of two core components: the humidity condensate membrane aerated biological reactor (HC-MABR) and the brackish water reverse osmosis (BWRO) units. This configuration is designed to treat low-strength wastewater—such as humidity condensate—efficiently, with minimal maintenance and energy use [16].

#### 2.1.1. Attributes of the Humidity Condensate Membrane Aerated Biological Reactor

**Figure 2:**
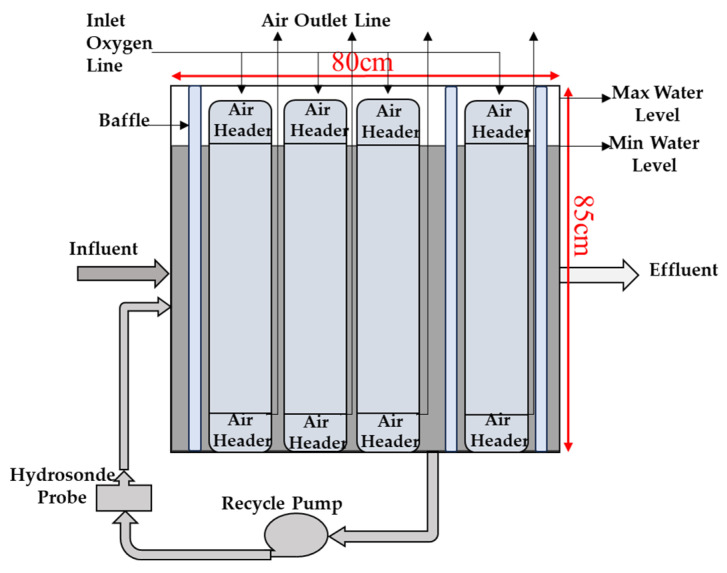
Humidity condensate MABR schematic (adapted from Hooshyari et al. [16]).

The humidity condensate membrane-aerated biological reactor (HC MABR) was engineered to support a treatment capacity of up to 26 L (13 crew) per day (Figure 2). With an average nominal hydraulic retention time (HRT) of seven days The MABR has been previously described in detail but is summarized here for convenience. The total and minimum wet volumes of the reactor were calculated as 238 L and 169 L, respectively. The reactor contained four independent siloxane membrane aeration modules, each equipped with dedicated inlet and outlet air headers. Each module consisted of 374 hollow, non-porous siloxane tubes with an internal diameter of 0.245 cm and an external diameter of 0.55 cm [16]. Compressed oxygen was delivered to all four modules at a rate of 100 mL/min via Masterflex mass flow controllers (model number 32907-55) (DWYEROMEGA at Michigan City, IN, USA). The effective membrane-specific surface area of the system was approximately 100 m^2^ per m^3^ of reactor volume. Internal mixing was maintained by a Laing LHB08100092 recirculation pump (Plumbers Supply Co. at Louisville, KY, USA) operating at a flow rate of 22 L/min. During operation, wastewater was introduced into the reactor continuously as it would be produced in a PGH.

#### 2.1.2. Attributes of Brackish Water Reverse Osmosis (BWRO) Unit

**Figure 3:**
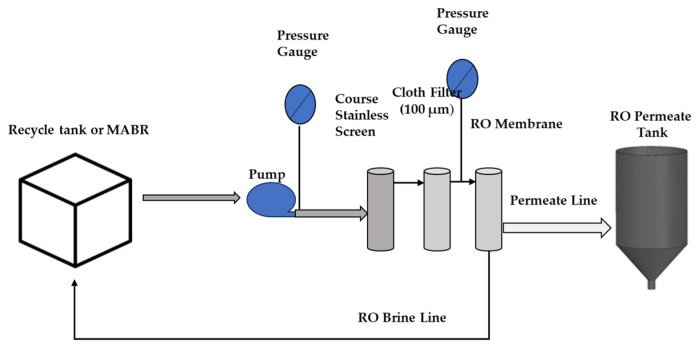
Brackish water reverse osmosis (BWRO) Unit (adapted from Hooshyari et al. [16]).

As illustrated in Figure 3, the brackish water reverse osmosis (BWRO) unit consisted of a 115-liter conical recycle tank (United States Plastic Corp, Lima City, OH, USA), a ClearPath MAP V2 recycle pump (Fluid-O-Tech International at Plantsville, CT, USA), a coarse stainless-steel screen (PRMFILTRATION, Butner, NC, USA), a cloth prefilter module (polypropylene fiber, 100 µm) (Clear 20 Inc., Northbrook, IL, USA), a BWRO treatment unit, and a 115-liter conical permeate collection tank. The system was designed to facilitate batch-mode operation at 3.45 bar (50 lb/in^2^) and enable continuous monitoring of flow and filtration performance. The BWRO unit employed a commercial Genuine DuPont FilmTec Element (BW60-1812-75) membrane module with a membrane area of 0.67 m^2^ (Fresh water Systems at Greenville, SC, USA). These membranes are a classic spiral-wound configuration, consisting of multiple membrane sheets wrapped around a central permeate tube with feed spacers between membrane layers and permeate carriers to collect treated water and guide it to the center tube.

#### 2.1.3. Humidity Condensates Stream Composition and Feeding Regime

Humidity condensate (HC) was made daily from concentrated stock solutions (Table 1). The HC composition was based on analysis of ISS samples and adapted from Muirhead et al. [5]. The HC has a DOC concentration of 90–120 mg/L, TN of 19–40 mg/L, TDS of 170–230 mg/L, COD of 190–240 mg/L, and BOD of 110–140 mg/L.

### 2.2. BWRO Treatment Process Configuration Type

#### 2.2.1. Configuration 1

In configuration 1 (Figure 1a), the HC MABR was loaded with 8 L of humidity-condensate per day, corresponding to the output of a four-person crew. The brackish water reverse osmosis (BWRO) system processed the MABR fluid at a constant inlet pressure of 3.45 bar (50 psi). During operation, the flow rate through the RO membrane module varied based on cumulative membrane operation time, ranging from 4.2 to 2.5 L per hour. This corresponds to a flux range of approximately 6.0 to 3.8 L h^−1^ m^−2^. A 115-liter conical tank was designated for permeate collection, while the brine stream was recirculated directly back into the HC MABR, enabling complete concentrate recovery within the system.

The process operated in batch mode with each cycle scheduled every four days, equating to a batch volume of approximately 32 L. System parameters, including BWRO module pressure, permeate flow rate, and internal recycle rate, were continuously monitored, with operational data manually recorded at regular intervals to ensure consistency and evaluate system performance.

#### 2.2.2. Configuration 2

In configuration 2 (Figure 1b), the HC MABR was loaded with 26 L of humidity condensate per day, reflecting the output of a thirteen-person crew. MABR fluid was transferred in batches to the BWRO recycle tank and treated at a constant membrane inlet pressure of 3.45 bar (50 psi). The RO membrane flow rate varied with cumulative membrane use, ranging from 6.0 to 1.8 L per hour, corresponding to a flux range of approximately 8.9 to 2.7 L h^−1^ m^−2^.

Permeate was collected in a separate conical storage tank, while the brine was recycled to the feed tank. Each operational cycle continued until 90% of the influent volume was recovered as permeate. The remaining 10%, designated as brine, was removed for downstream processing or disposal. The system typically processed four to five days’ worth of MABR fluid per batch, depending on HC availability and system load. Key performance metrics—including RO module pressure, permeate production rate, and internal recycle rate—were monitored throughout the testing period. Manual data logging was performed regularly to ensure comprehensive tracking of system performance and to facilitate process evaluation.

### 2.3. Testing and Evaluation of Treatment System

Water samples for water quality analyses were collected from the humidity condensate (HC) stream and the final RO permeate three times per week throughout the experiment. MABR fluid samples were taken immediately after being transferred to the RO recycle tank to capture representative conditions. During treatment tests, pH and dissolved oxygen (DO) within the reactor were measured using a portable Hach H11d multiparameter pH/ORP meter.

All collected samples were filtered through 0.45 µm membrane filters and stored at 4 °C until subsequent analysis. Anion concentrations—including chloride (Cl^−^), nitrite (NO_2_^−^), nitrate (NO_3_^−^), phosphate (PO_4_^3−^), and sulfate (SO_4_^2−^)—were determined using a Dionex ion chromatography (IC) sodium carbonate/bicarbonate eluent with a 4 mm AS20 column and 4 mm suppressor. Total nitrogen (TN) and dissolved organic carbon (DOC) were quantified with a TOC-L CSH Shimadzu total organic carbon (TOC) analyzer. Before DOC analysis, samples were acidified and stored for a minimum of 24 h to ensure the removal of inorganic carbon. In addition, biological oxygen demand (BOD) and chemical oxygen demand (COD) tests were conducted weekly on the influent and MABR fluid of the MABR as well as on the RO permeate to evaluate water quality and treatment efficiency.

## 3. Results

### 3.1. Organic, Nutrient, and Inorganic Salt Removal in the HC MABR-BWRO System

#### 3.1.1. Configuration 1

**Figure 4:**
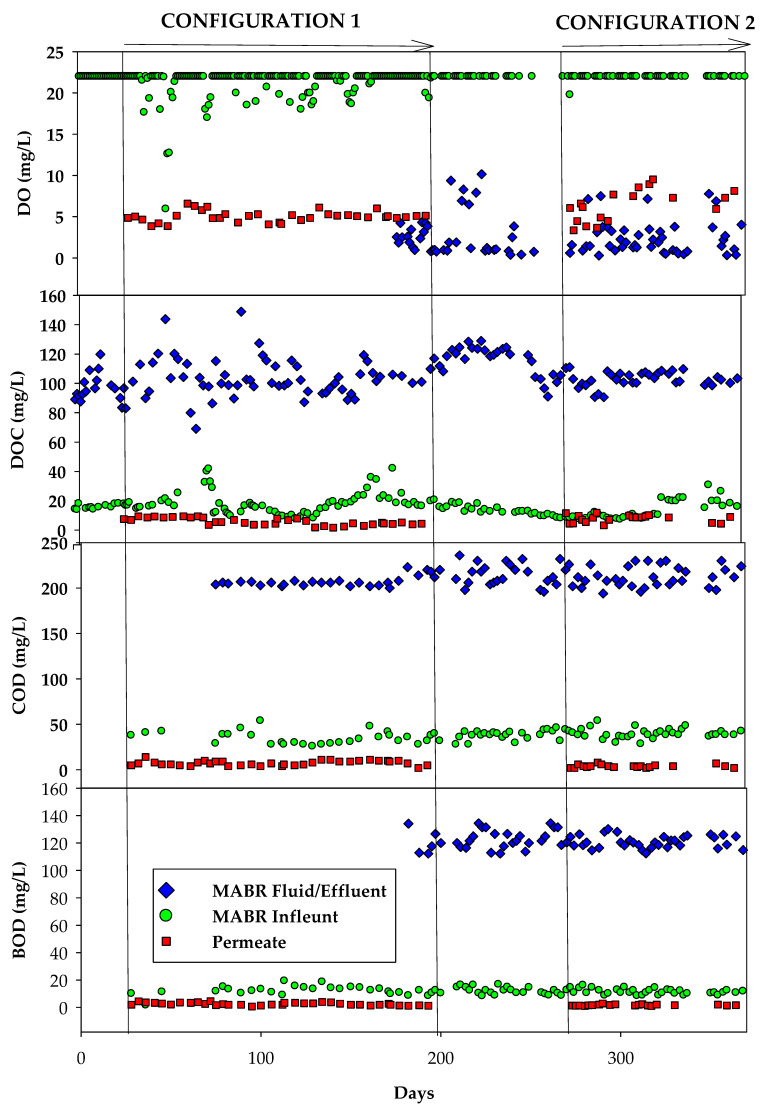
Assessment of organic matter removal performance in the integrated HC MABR and BWRO system.

The MABR reduced the influent DOC (80–140 mg/L), COD (202–230 mg/L), and BOD (105–130 mg/L) by greater than 80%, 85%, and 80%, respectively (Figure 4). The steady-state concentrations in the reactor were generally less than 20 mg/L, 40 mg/L, and 15 mg/L, respectively. This strongly supports that most degradable organic matter was oxidized. In addition, the elevated oxygen concentrations, near saturation (~20–22 mg/L), suggest that the system was not operating at capacity and was not oxygen limited. The HC MABR system DOC, COD, and BOD did not increase over time, despite that no brine was wasted during the BWRO process. RO Permeate DOC, COD, and BOD concentrations were very low (<7 mg/L, <10 mg/L, and <4 mg/L, respectively), and very stable with time. The OC carbon concentrations are near potable limits and indicate a stable solution with minimal biofilm formation potential. These results demonstrate the HC MABR’s capability to support high-efficiency organic removal under zero liquid discharge conditions, reinforcing its suitability for long-duration or resource-constrained treatment systems.

**Figure 5:**
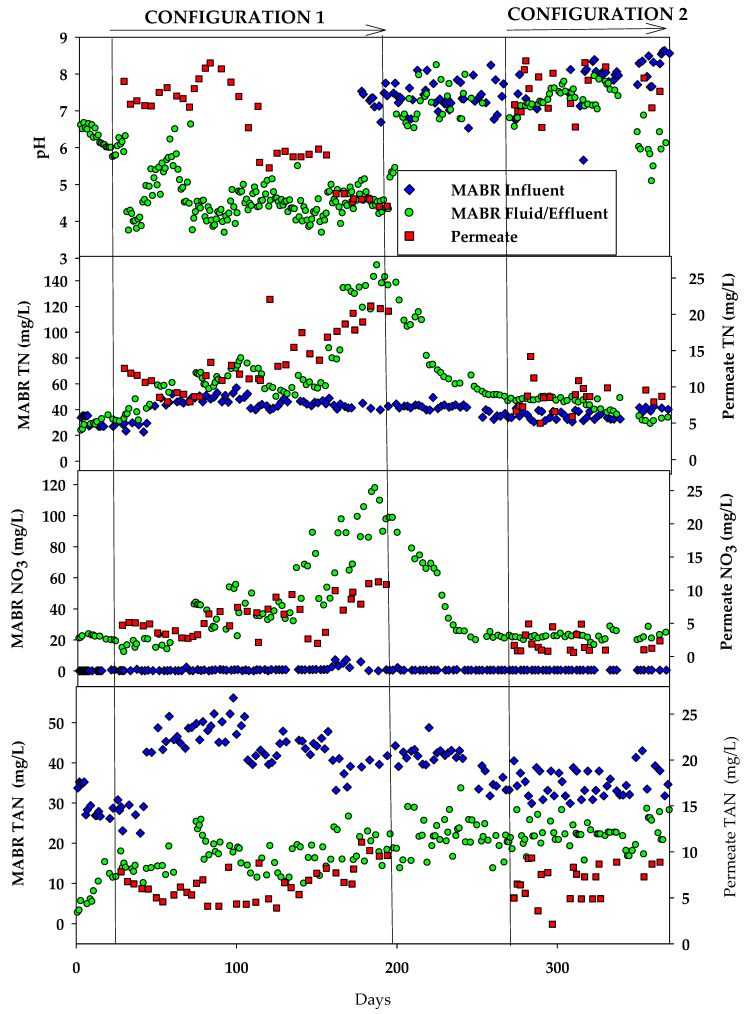
Assessment of nutrient removal in the HC MABR and BWRO treatment system.

The MABR system achieved consistent TAN oxidation, reducing influent TAN from ~50 mg/L to ~20 mg/L (Figure 5). The oxidation of TAN produced NO_3_^−^, the concentration of which increased throughout the test, reaching ~120 mg/L by the test cessation, due to production in the MABR and rejection by the RO system. The relative production for NO_3_^−^ and associated proton production, consumption of bicarbonate due to growth, and differential rejection of NO_3_^−^ compared to NH_4_^+^ resulted in a strong pH drop (<5 generally) in the MABR. The low pH through most of the test period likely limited TAN oxidation due to a lack of alkalinity to support growth. This has been observed in other wastewater habitation studies as well, in which an oxidation efficiency of 50–70% was generally observed [17,18,28].

In the permeate, TAN concentrations ranged from ~5–10 mg-N/L, generally increasing throughout the test period. Similarly, NO_3_^−^ concentrations increased from ~5 mg-N/L initially to ~10 mg-N/L by the end of the test period. The relative rejection of NO_3_^−^ increased from ~75% to >90%, but TAN rejection decreased from ~75% to ~50% throughout the test period. The decrease in TAN rejection may be due to NO_3_^−^ transport through the membrane, which forced increased TAN passage to maintain charge neutrality. The increase in TAN concentrations in the permeate over the test run also caused the pH of the permeate to decrease from ~7 to ~4 throughout the test period, due to NH_4_^+^ (the dominant form in MABR) disassociation to NH_3_ and H^+^. The lower pH of the permeate is beneficial for space habitation systems that use ion exchange to remove trace anions and cations, as it reduces the bicarbonate concentration, which can consume exchange capacity.

**Figure 6:**
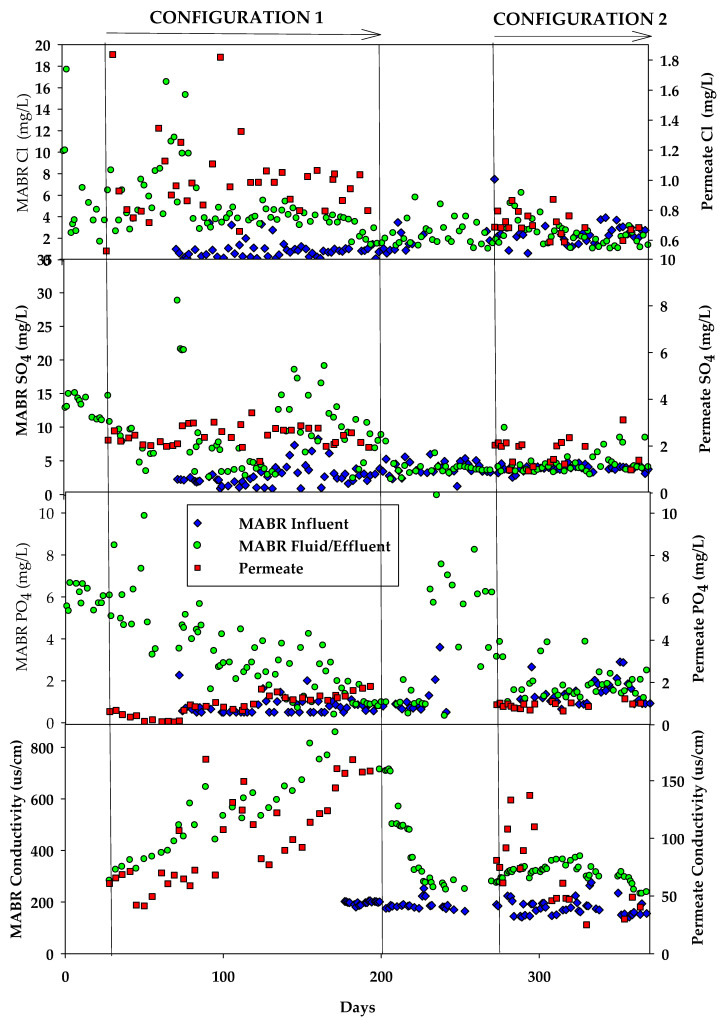
Assessment of inorganic ion and conductivity in the integrated HC MABR and BWRO treatment system.

The influent HC has very low concentrations of Cl^−^, SO_4_^2−^, and PO_4_^3−^ (<2 mg/L). Concentrations of the species in the reactor were higher than influent concentrations due to residuals from past testing and generally decreased throughout the test period. The conductivity of the MABR fluid increased from ~200 µs/cm, similar to the conductivity of the influent feed, to >800 µs/cm after processing ~1000 L of HC (Figure 6), driven by the accumulation of ions rejected by the RO system and retained in the reactor. The permeate conductivity increased throughout the test period from ~50 µs/cm to 180 µs/cm at the end of the test. NO_3_^−^, TAN, and H^+^ were the dominant measured species in the permeate. Other anions such as SO_4_^2−^, Cl^−^ and PO_4_^3−^ were generally less than 2 mg/L throughout the test period. On a charge equivalent basis, the permeate NO_3_ and NH_4_ each constituted 45% of the total permeate conductivity, H^+^ 6%, and all other species combined <4%.

**Figure 7:**
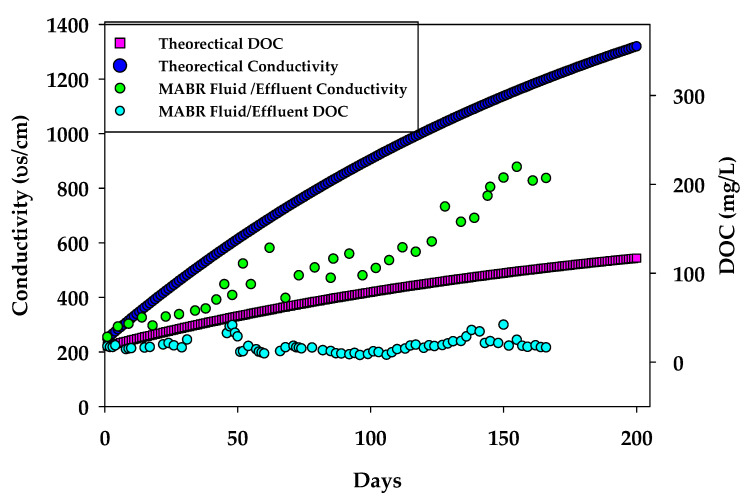
Theoretical simulation assessment of DOC and conductivity in the HC MABR build-up system.

Simulation results (Figure 7) evaluating the DOC and conductivity based on the assumption of a 90% rejection indicate that conductivity in the MABR gradually increased over time and approached the influent value (~200 µS/cm) after approximately 100–120 days. This reflects the conservative nature of ionic species, which are not biologically removed and accumulate in the system due to the lack of brine discharge. In contrast, DOC concentrations remained stable and did not accumulate, despite continuous loading from the influent. The MABR maintained DOC levels near the initial value (~18 mg/L), demonstrating effective biodegradation of the influent organic matter. This suggests that the organic load consists largely of slowly degradable compounds and that the biofilm remains active and capable of maintaining treatment performance over extended periods without requiring brine removal.

#### 3.1.2. Configuration 2

In configuration 2, in which there was an external recycle tank, the MABR was loaded at a much higher rate (26 L/day) than in configuration 1 (8 L/day). Even at this increased loading rate, DO concentrations in the MABR remained consistently near saturation (~22 mg/L). DOC, COD, and BOD influent concentrations were constant through the configuration test and very similar to influent concentrations throughout configuration 1 testing (Table 2). MABR fluid DOC, COD, and BOD concentrations were also very similar (20–40 mg/L, 30–50 mg/L, and 15–20 mg/L, respectively) to MABR fluid concentrations in configuration 1 testing (Figure 4). Similar concentrations between configurations, even though the loading was 3.25 times greater in configuration 2 and in configuration 1 no brine was removed, suggest that the reactor was not kinetically limited (e.g., could be smaller and achieve the same performance) and that a portion of the residual DOC is degradable over longer time periods. The permeate water quality, as reflected in DOC, COD, and BOD concentrations (DOC < 5 mg/L, COD < 5 mg/L, BOD < 3 mg/L), was also very similar to permeate concentrations in configuration 1 testing. These low concentrations confirm the effectiveness of the combined biological–membrane treatment and the suitability of the system for long-term water recycling. Particularly with respect to producing a biologically stable permeate.

Total ammoniacal nitrogen (TAN) in the MABR influent varied between 30 and 45 mg-N/L. MABR fluid TAN concentrations were variable but generally ~20 mg/L which was similar to concentrations at the end of configuration 1 but higher than those during the initial period of configuration 1 (Figure 5 and Table 2). NO_3_^−^ concentrations in the MABR fluid remained relatively stable at ~20 mg-N/L and similar to the initial NO_3_^−^ concentrations during configuration 1 prior to when rejection of NO_3_^−^ by the RO caused an increase in concentrations, similar to the period of operation before configuration 1. Permeate NO_3_^−^ and TAN concentrations (<5 mg-N/L and 5–10 mg-N/L, respectively) were stable during configuration 2 testing. MABR fluid and permeate pH were stable between 6.5 and 7.5. The higher pH in the reactor is likely due to the lower overall TAN oxidation efficiency. The higher MABR fluid pH would have caused increased alkalinity and higher ratios of NH_3_/NH_4_ compared to configuration 1 and is likely responsible for the higher permeate pH.

MABR fluid conductivity remained stable and consistently below 250 µS/cm, with limited fluctuation. Permeate conductivity was also stable and <100 µS/cm throughout the test. Chloride (Cl^−^), sulfate (SO_4_^2−^), and phosphate (PO_4_^3−^) were all very low <3 mg/L and similar to configuration 1 testing (Figure 6 and Table 2).

### 3.2. Permeate Flux

#### 3.2.1. Configuration 1

**Figure 8:**
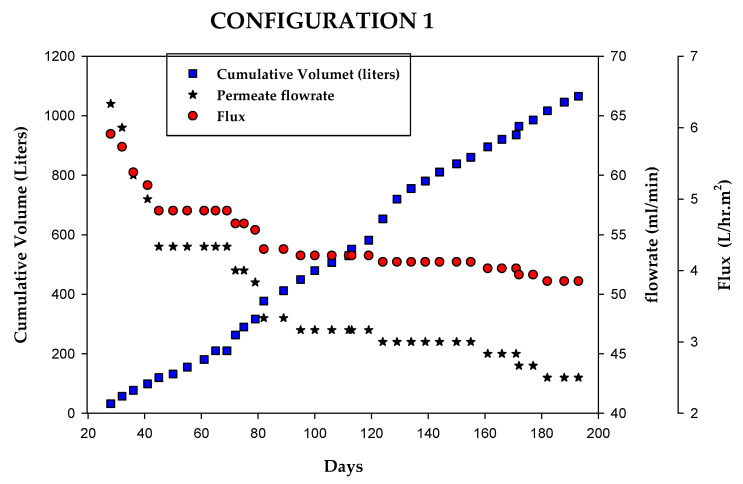
BWRO system overall performance test: RO brine/reject recycled to MABR.

During configuration 1 testing, the reverse osmosis (RO) system treated approximately 1100 L of fluid, equivalent to 550 crew-days, over a 175-day operational period (Figure 8). Notably, the membrane utilized had previously been employed to treat biologically pretreated space habitation wastewater. As a result, the membrane exhibited approximately 50% of the flux performance of a new membrane; however, its operational capacity remained well above the required throughput. The permeate flow rate declined from approximately 65 mL/min to 55 mL/min after processing only ~100 L and further decreased to ~45 mL/min over the remainder of the test period, following a non-linear decline trend. The operating pressure was maintained consistently at 3.45 bar (50 psi), yielding a flux range of 6.3 to 3.7 L per hour per square meter (L h^−1^ m^−2^).

Although the test was terminated due to elevated permeate conductivity, the RO system retained the capability to process the daily influent humidity condensate (HC) volume of 8 L in less than 3.2 h. The observed decline in permeate flow rate is attributed primarily to membrane fouling, as the relatively modest increase in conductivity would not have significantly elevated osmotic pressure. This suggests that organic or biological foulants were the dominant contributors to performance degradation.

#### 3.2.2. Configuration 2

**Figure 9:**
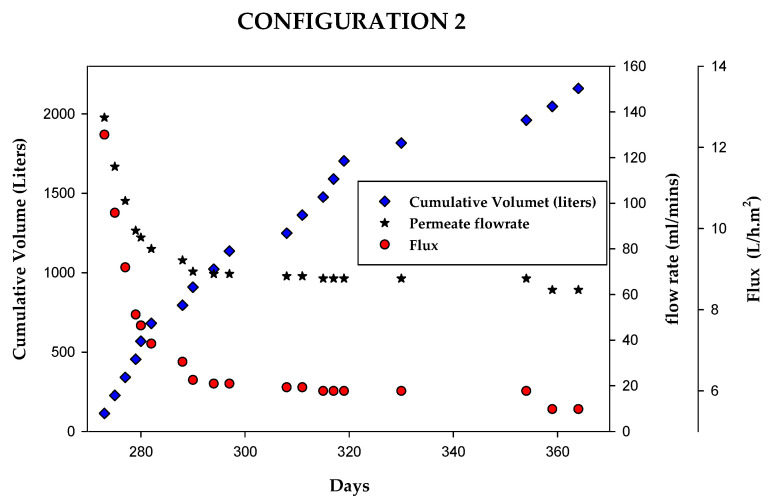
BWRO system overall performance test: RO brine/reject recycled to separate RO feed/concentrate recycle tank.

In configuration 2 (Figure 1b), the HC MABR was loaded with 26 L of humidity condensate per day, corresponding to the output of a thirteen-person crew. The brackish water reverse osmosis (BWRO) system processed approximately 2160 L of the MABR fluid at a constant inlet pressure of 3.45 bar (50 psi) (Figure 9). During operation, the flow rate through the RO membrane module varied based on cumulative membrane operation time, ranging from approximately 140 mL/min to approximately 69 mL/min after 1000 L of the produced water and then slightly decrease to approximately 62 mL/min over the remaining operating period (approximately 1100 L of treated water). This corresponds to a flux range of approximately 6.0 to 12 L h^−1^ m^−2^.

It should be noted that when the test was terminated, the RO system could still process the daily influent HC volume (37.2 L) in less than 10 h. While it is not advisable to extrapolate to predict the total membrane life, it does appear that in configuration 2 the total life of the membrane could be substantially longer than the tested 1080 crew-days of processed HC.

## 4. Discussion

The results of this study demonstrate that both MABR-RO configurations offer substantial performance advantages for closed-loop wastewater treatment in spaceflight and terrestrial analogs. However, translating these experimental successes into viable, long-term solutions requires addressing a series of practical, operational, and systems-integration challenges.

Brine Management and Long-Term Stability:

Configuration 1, which operated without brine wasting, successfully reduced DOC, COD, BOD, and conductivity over approximately 500 crew-days. However, the absence of brine removal presents a critical limitation. Accumulation within the MABR reactor led to a progressive increase in conductivity and poses a risk of osmotic pressure buildup, membrane scaling, and loss of treatment efficiency. Although strategies such as periodic brine discharge (~10% of daily influent volume) or batch bypass to a distillation subsystem could mitigate these effects, they introduce additional complexity, power demands, and system mass. These tradeoffs must be evaluated in the context of mission duration, logistics constraints, and subsystem redundancy.

Scalability and Modular Design:

Configuration 2 demonstrated operational stability over 1000+ crew-days while supporting high daily loading (26 L/day, equivalent to 13 crew members). While this validates the scalability of the system up to a certain threshold, further expansion will require proportional increases in membrane area, oxygen delivery, and reactor volume. This poses design and integration challenges, particularly for spacecraft with constrained volume, thermal control, and power budgets. Future designs could explore modular architectures that allow parallel operation of multiple smaller units to support larger crews or redundancy while maintaining manageable form factors.

Operational Risks and System Robustness:

Membrane performance degradation remains a key operational risk. In both configurations, a decline in flux was observed over time. While part of this can be attributed to biofouling or membrane aging, it also reflects potential vulnerability to variable loads or upstream biological shifts. These risks highlight the need for adaptive control strategies and autonomous system health monitoring. Cleaning protocols, membrane regeneration methods, and fouling-resistant materials should be evaluated for long-term missions, especially under microgravity, where standard hydraulic and flushing operations are constrained.

Maintenance Requirements and Consumables:

Long-duration missions require a clear understanding of component durability and resupply needs. While the projected annual consumable mass (<1 kg/year/crew) for oxygen and membranes is low relative to traditional systems, membrane longevity and bioreactor stability remain critical. Preventive maintenance schedules, in situ diagnostics, and onboard regeneration capabilities would improve system reliability and reduce astronaut intervention. These requirements must be factored into overall environmental control and life support system (ECLSS) planning, particularly for missions exceeding 1 year in duration.

Compatibility with Existing ISS Systems:

One of the potential advantages of configuration 1 was its lower permeate pH (4–6), which could reduce bicarbonate loading on ISS ion exchange beds and extend their life. However, this also introduces possible challenges with materials compatibility and downstream subsystem integration, particularly for systems calibrated for neutral pH influent. pH adjustment or buffering may be necessary prior to final polishing. Furthermore, compatibility with existing brine stabilization hardware, such as the UPA/Brine Processor Assembly (BPA), requires flow synchronization, ion balance alignment, and harmonized operational schedules to avoid overloading or underutilization.

System Integration Outlook:

The integration of MABR-RO into future spacecraft or planetary habitats must consider the entire life support ecosystem. Factors such as thermal regulation, oxygen demand, bioreactor startup kinetics, and control system architecture must align with existing spacecraft standards. Moreover, the automation, fault tolerance, and remote diagnostics of the treatment system will be critical for deployment in crewed deep-space missions or lunar/Martian surface habitats.

According to (Table 3), configuration 2 demonstrated superior performance across all evaluated metrics, particularly in conductivity reduction, permeate pH stability, and long-term operational resilience. DOC removal in both configurations was highly effective, achieving levels comparable to or better than the ISS WPA. Notably, the low consumables requirement and moderate storage volume further support system suitability for long-duration missions and spaceflight applications. Configuration 1, while functional, exhibited lower conductivity rejection and less favorable pH control, underscoring the importance of system configuration in optimizing water quality and process stability.

## 5. Conclusions

Additional work is required to demonstrate and evaluate configuration 1 as a long-term operating system to support space habitation systems. For configuration 2, it is possible to evaluate system size and consumable mass. Given that the MABR was able to process a load of 13 crew/day, for projected missions with a crew of 4, the MABR size would be no larger than ~50 L (~6-day residence time). Based on the above, if the recycle tank has a volume of 8 L, the total storage volume would be <60 L. For comparison, on the ISS, the water processing assembly storage tank has a volume of ~50 L. In terms of consumables, even assuming the RO membrane can only process ~2000 L, the volume demonstrated in this study, the total consumable mass consisting of O_2_ (0.00024 kg/kg of processed water) and RO membrane (0.25 kg per 2100 kg of processed water) would be <1 kg per year for a crew of four.

Overall, the results suggest that a hybrid water processing system, which replaces a passive storage tank for HC with an engineered bioreactor that is coupled to a small-scale (<139 kg total system mass) low-pressure (<50 lb/in^2^) RO system, could provide near potable water for extended periods (>1000 crew-days). The increased system mass would be more than offset by reducing IX bed consumption (~25% of consumption W/O RO), and the system would eliminate downstream biofouling, a significant issue observed on the ISS. Additional work should be conducted to further explore the optimized configuration and extended RO life as well as demonstrate the total mass savings.

## Figures and Tables

**Table 1:** Humidity condensate Ersatz for ground testing of water processors [5].

SI NO	Ingredient	Concentration mg/L
1	Ethanol	67
2	Propylene Glycol	27
3	Methanol	6.5
4	Benzyl Alcohol	15
5	Ethylene Glycol	4.5
6	Acetone	2.6
7	Caprolactam	2.3
8	2-Propanol (Isopropanol)	1.0
9	Benzoic acid	2.0
10	2-Phenoxyethanol	2.0
11	2-(2-Butoxyethoxy) ethanol	2.0
12	N, N-Dimethyl acetamide	0.9
13	Diethylphathalate	1.2
14	Trimethylsilanol	0.41
15	Acetaldehyde	0.16
16	Formaldehyde	0.081
17	Zinc Acetate	15
18	Nickel Acetate	5.9
19	Ammonium Bicarbonate	198.4
20	Ammonium Acetate	14.8
21	Ammonium Formate	2.9
22	Ammonium Fluoride	1.4
23	Monopotassium Phosphate	0.6
24	Calcium Bicarbonate	1.4
25	Sodium Bicarbonate	0.3

**Table 2:** A comparative summary table presenting average values and standard deviations of influent and permeate quality—specifically for DOC, TN, and conductivity—was added for each configuration test. This table enables direct evaluation of removal performance across system configurations.

Test Type	Configuration 1					
	Average	Standard Deviation	Average	Standard Deviation	Average	Standard Deviation
	Influent		Effluent		Permeate	
**DOC (mg/L)**	105	12.2	15.9	6.83	6.53	2.64
**TN (mg/L)**	39.6	7.13	64.4	29.9	12.1	4.38
**Conductivity (mg/L)**	188	24.7	398	166	93.6	38.6
Test Type	Configuration 2					
	Average	Standard Deviation	Average	Standard Deviation	Average	Standard Deviation
	**Influent**		**Effluent**		**Permeate**	
**DOC (mg/L)**	99.1	3.91	22.1	5.22	6.06	2.54
**TN (mg/L)**	39.2	3.24	32.6	2.58	8.73	0.815
**Conductivity (mg/L)**	183	29.9	246	28.3	39.8	9.53

**Table 3:** Comparative table.

Metric	Config 1 Final Permeate	Config 2 Final Permeate	ISS WPA Final Produced Water [29,30]	Graywater MABR-RO Final Permeate [16]
DOC Removal (%)	~94%	~94%	~98%	~90%
Conductivity reduction (%)	Moderate (~50%)	High (~78%)	~60%	~60%
pH of permeate	4–6	6–8	6.5–8.5	~7
Operational days (crew-days)	~500	>1000	Continuous	200–1000
Biofouling control	High	High	None	Very High
Consumable mass (Kg/year/crew)	<1	<1	N/A	~2–3

## Data Availability

Data are available upon request.

## Glossary of Acronyms

BWRO	brackish water reverse osmosis
DOC	dissolved organic carbon
ECLSS	environmental control and life support system
GW	greywater
HC	humidity condensate
HRT	hydraulic retention time
ISS	international space station
NASA	National Aeronautics and Space Administration (USA)
MABR	membrane-aerated biological reactor
PGH	partial gravity habitation
RO	reverse osmosis
TAN	total ammoniacal nitrogen
TDS	total dissolved solids
THC	temperature and humidity control
TN	total nitrogen
TOC	total organic carbon
WPA	water process assembly
WRS	water recovery system
